# Editorial: Lifestyle, biological risk factors for non-communicable diseases in the midst of social inequalities and COVID-19

**DOI:** 10.3389/fnut.2023.1235752

**Published:** 2023-07-10

**Authors:** Kotsedi D. Monyeki, Andre Pascal Kengne, Benedicta Ngwenchi Nkeh-Chungag, Han C. G. Kemper

**Affiliations:** ^1^Department of Physiology and Environmental Health, University of Limpopo, Sovenga, South Africa; ^2^Non-Communicable Diseases Research Unit, South African Medical Research Council and University of Cape Town, Cape Town, South Africa; ^3^Department of Biological and Environmental Sciences, Faculty of Natural Sciences, Walter Sisulu University, Mthatha, South Africa; ^4^Health and Physical Activity, Amsterdam University Medical Centre, VUmc, Amsterdam, Netherlands

**Keywords:** non-communicable disease (NCD), COVID-19, social inequalities, risk factors, lifestyle

Poverty involves a variety of human deprivations, including those related to food consumption, education, security, health, dignity, and a decent job (1, 2). Relative poverty is defined as a person's failure to reach a minimal quality of life in comparison to others in the same location and time (3), while subjective poverty is a personal perspective of financial or material position (4). Poverty is the primary underlying cause of reduced food security and quality and quantity of consumed food, which exposes people to low dietary diversity and increases their risk of developing non-communicable diseases (NCDs).

The NCD profile is changing rapidly over time amongst rural South African populations due to COVID-19, poverty, and other psychosocial factors. The South African Development Plan highlights important recommendations for a 28% reduction in the prevalence of NCDs by the year 2030 in line with the requirements to meet the Sustainable Development Goal (SDG) target (5). Similarly, the World Heart Federation has set the year 2025 by which to have reduced NCD prevalence by 25% (6). However, the onset of the COVID-19 pandemic in early 2020 has already likely further compromised the efforts toward attaining the national and global targets. Some individuals and families have been pushed back to extreme poverty in low socioeconomic contexts. Therefore, efforts to tackle the triple burden of NCDs, poverty, and COVID-19 are urgent. Unfortunately, low literacy levels impact the triple burden of disease, and recommended lifestyle changes remain extremely low among individuals, families, and communities. Health promotion initiatives by medical experts, academics, and scholars are crucial if one wants to be successful in changing an individual's lifestyle. The community must look for novel and creative solutions to address demographic challenges such as overweight/obesity, hypertension, physical inactivity, smoking cessation, and alcohol abuse to maintain healthy-living lifestyles. Furthermore, to work toward alleviating the burden of NCD today, it is crucial to have a thorough understanding of the CVD risk factors, such as behavioral, clinical, and sub-clinical factors, as well as the resulting target organ damage.

Health professionals, academics, scholars, and community members gathered in the University of Limpopo and Kitty Village, Lephalale, during the period from 22 to 24 November 2022 from all parts of the world under the unique light of the 3^rd^ Ellisras Longitudinal Study and Other Non-Communicable Diseases International Conference. The theme of the conference was “multi-morbidity, poverty, and COVID-19- are we winning?” New knowledge of NCDs acquired through laboratory and field work efforts and dedication was imparted. The series in this e-book Research Topic, among others, include a Research Topic of articles developed from selected abstracts presented at the 3rd ELSONCDIC and other sources such as Frontiers in Nutrition and Frontiers in Public Health.

In this Research Topic, there are 14 papers covering the above-mentioned topic. Four articles covered the section on **lifestyle choices and non-communicable disease risk factors**. A systematic review and meta-analysis reported a positive correlation between cumulative cortisol activity and adiposity-related complications, with cortisol levels being the only predictor of long-term stress among children. Furthermore, Ma et al. did not report any significant association between body mass index (BMI)/BMI z-score and the concentration of morning salivary cortisol and total daily cortisol output. Sekgala et al. (a) reported that the chances of developing metabolic syndrome (MetS), aberrant HDL-C, triglycerides, and hypertension were enhanced by alcohol consumption, sugar-sweetened drinks (SSBs), fried foods, and snacks from street food (SF) vendors. Furthermore, the body fat percentage and Clínica Universidad de Navarra-Body Adiposity Estimator (CUN-BAE) were the best discriminators of MetS, followed by the waist circumference, waist-to-height ratio, BMI, and the body roundness index (BRI) as the last discriminator. All the anthropometric measures demonstrated outstanding discriminatory abilities for predicting MetS, with c-statistics often larger than 0.8 [Sekgala et al. (b)]. Finally, gender disparity was detected in alcohol use by Mmereki et al., whereby adolescents aged 15–17 years and 18–21 years showed greater alcohol consumption than those aged 13–14 years.

Three manuscripts covered the section on **the COVID-19 pandemic and lifestyle**. Patients who previously only complied partially with treatment instructions before COVID-19 and who had an unstable disease condition were more susceptible to pandemics and epidemics and may require special care if such widespread outbreaks recur in the future (Jiao et al.). Participants affected by food insecurity (FI) had greater anxiety about poverty, COVID-19, and the health effect of FI on their lives. Additionally, the relationship between financial and physical wellbeing was found to be mediated by FI but not mental quality of life (QOL) indicators (Karam et al.). Male participants were most likely to follow an unhealthy lifestyle, such as drug use and smoking, while female participants were the most likely to be physically inactive (Sultana et al.).

**The poverty and risk of NCDs section was covered in three manuscripts**. It was found that a person's physical health is greatly impacted by the length of their subjective poverty, particularly if they live in a rural area (Cao et al.). Householder smoking had a significant impact on the household's likelihood of living in poverty, while having an NCD had a beneficial mediation effect (Yang et al.). Children from low-income backgrounds were shorter and leaner than those from high-income backgrounds, but they maintained their lean mass, which is an important trait for male reproduction. Due to lesser energy reserves and the avoidance of cardiometabolic expenses, their immune systems may have been compromised (Wells et al.).

The section on **nutrition and dietary patterns included two manuscripts**. On an interpersonal level, the best source of breastfeeding support was found to be mostly in the family. However, family interference also serves as a roadblock to breastfeeding. On the community level, family beliefs and practices are common among mothers. However, different traditional beliefs and societal and cultural norms divide them in the promotion or obstruction of breastfeeding. Intervention programs should concentrate on behavior modification to inform and prepare mothers to overcome controllable obstacles (Seabela et al.). Teenagers in Zambia were found to prefer four main dietary patterns: snacking, which includes eating sweets and snacks; vegetarianism, which includes eating pulses, fish, and vegetables; health consciousness, which includes eating fruits and eggs; and traditional, which includes eating cereal and meat. Teenagers also adhered to rather less common and healthier vegetarian and health-conscious dietary habits (Mukanu et al.).

Only one article was available in the section on the use of **a questionnaire to achieve valid estimates of any health measure, HIV antiretroviral therapy, and increased endothelial biomarkers**. Decision-makers and researchers in the health profession should be mindful that question-order biases can alter results from two questionnaires with questions that have the same words but a different order or clustering, rendering the comparison of the results from the two questionnaires invalid. The results imply that when evaluating the scale of a subjective health-related phenomenon exposed to significant ambiguity (e.g., the perceived danger of occasional and frequent drug use), the responses may greatly depend on the question ordering when using two or more consecutive or adjacent items (Pérez-Romero et al.). After adjustments for CVD risk factors, HIV status was linked to higher levels of endothelial dysfunction biomarkers when compared to a HIV-negative control (Hanser et al.).

## Summary

Urbanization is associated with increased changes in lifestyle and diet. Therefore, it is hypothesized to contribute to the increase of CVD worldwide, especially during the COVID-19 pandemic phase. Furthermore, susceptibility to hunger, food insecurity, poverty, negative socioeconomic factors, and unhealthy lifestyles have also contributed to the global increase in CVD prevalence. Lifestyle changes that encourage the cessation of risky behavior should be promoted and will eventually benefit individuals and communities. Influential lifestyle changes begin with the acquisition of accurate knowledge through personal interaction with health experts, scholars, and academics who disseminate health information to different sectors of the community.

## Author contributions

KM wrote the first draft and sent it to AK, BN-C, and HK for critical review and input. The article's submission was reviewed and approved by all the authors. All authors contributed to the article and approved the submitted version.
